# Relations between a social emotional learning (SEL) program and changes in resilience, self-esteem, and psychological flourishing in a youth sample

**DOI:** 10.1007/s44192-025-00173-x

**Published:** 2025-03-27

**Authors:** Kayla Brill, Claire McGuinness, David Nordstokke

**Affiliations:** School and Applied Child Psychology, University of Calgary, Calgary, Canada

**Keywords:** Social emotional learning (SEL), Resilience, Youth development, Strengths-based education

## Abstract

The HEROES program is a Social Emotional Learning (SEL) initiative designed to foster resilience, self-esteem, and flourishing in youth through strengths-based, experiential learning. This study evaluated the program’s impact among Grade 7 and 8 students (N = 87) in rural Alberta, Canada, measuring changes at four time points: pre-intervention, post-intervention, 2 month follow-up, and 5 month follow-up. Resilience was assessed using the Connor-Davidson Resilience Scale (CD-RISC-10), while self-esteem and flourishing were measured with the Rosenberg Self-Esteem Scale (RSES) and the Flourishing Scale (FS), respectively. Repeated-measures ANOVA, using gender as a grouping variable, showed a significant increase in resilience from pre- to post-intervention, which was maintained through 2- and 5 month follow-ups, suggesting sustained program effects. While no significant changes were observed in self-esteem or flourishing scores, minor positive shifts occurred. No gender differences were present across the study variables. These findings indicate that the HEROES program is effective in promoting resilience in youth but may require additional elements to impact self-esteem and psychological flourishing meaningfully. This study contributes to SEL literature by highlighting the potential of school-based interventions to improve youth resilience, with implications for expanding such programs in educational settings. Future research should examine the program's long-term effects and explore how facilitators might optimize outcomes across diverse populations.

## Introduction

There is an unprecedented increase in childhood and adolescent mental health challenges [1–3]. Specifically, 81% of youth reported having been negatively impacted by stress, with one in five children in Canada having a mental health problem [1]. Children, like adults, are confronted with a variety of circumstances that necessitate coping and adaptation; however, due to their limited life experiences, resources, and knowledge, this population may be at risk for developing cognitive, emotional, and behavioural problems [4]. Children and adolescents who suffer from persistent stress and other mental health challenges are more likely to experience mental health challenges in adulthood, as well as academic and interpersonal difficulties [1, 5, 6]. Altogether, this emphasizes the need to identify strategies to prevent mental health challenges and enhance student well-being.

Given its importance, research has focused on protective factors in relation to student well-being. This includes individual characteristics such as social and emotional skills (e.g., communication, problem-solving, and relationship skills), a sense of optimism about the future, and resilience [7]. Previous research has demonstrated that resilience serves as a protective factor against psychological distress among students, with resilience training reducing symptoms of both depression and anxiety [8, 9]. As such, there has been a greater emphasis on prevention and early interventions to improve youth resilience to prevent adversities from arising or continuing later in life and to assist those who are at risk so that they may succeed in school. This proactive approach not only targets mental health challenges but also fosters a supportive environment that encourages positive relationships, skill development, and community engagement among young people [10]. To achieve this, it is essential to develop programs that involve parents, educators, and community leaders, as they play an important role inbuilding a comprehensive support system that empowers youth to navigate life's challenges effectively [11]. By integrating resources and fostering collaboration among various stakeholders, these initiatives can create a sustainable framework that not only addresses immediate concerns but also promotes long-term well-being and success for children and adolescents [12, 13].

### Resilience

In general, resilience is defined as the process and outcome of successfully adapting to difficult or challenging life experiences through aspects of flexibility and adjustment [14]. According to Masten, resilience involves one being able to recover from challenges, to function as well as before and move forward with their lives [15]. Those who are resilient are more able to cope with, or adapt to, stress and challenges [16]. They learn from experiences of being able to effectively manage in one situation, making them better able to overcome challenges in future situations [17]. Children who show more empathy and perceived social support may be more resilience [18]. Resilience in children has been associated with effective communication skills [19], improved problem solving skills [20], are driven to achieve goals [21], are hopeful about the future [22], have a secure relationship with one or more adults [23], and they feel safe in their communities [24]. Ultimately, promoting resilience is connected to better mental health outcomes [24].

Much of the resilience research is focused on supporting resilience through interventions to promote or protect mental health and development [25]. There are several widely replicated protective factors that are typically targeted in these resilience-building interventions to improve mental health outcomes. These factors include instilling positive relationships with caring adults, promoting effective parenting, enhancing problem-solving and self-regulation skills, increasing perceived efficacy and control, achievement motivation, creating positive relationships (e.g., friendships), maintaining spirituality, having beliefs that life has meaning, and having effective teachers and schools [26, 27]. Through fostering these factors, resilience building programs have been shown to promote positive mental health outcomes in students (i.e., reducing anxiety and depression), improve academic performance, and help students build positive relationships with others [28, 29].

### Social emotional learning (SEL)

Social emotional learning (SEL) interventions have shown promise in fostering resilience among youth across diverse contexts [30–32]. For example, the “Forward with Peers” program, a 10 week school-based program that aims to create a safe place to further culturally adaptive social and emotional learning and build students’ toolset to achieve their academic, professional, and personal goals, evaluated among Arab immigrant and refugee adolescents in the Detroit Metropolitan Area, demonstrated statistically significant improvements in perceived social support and marginal gains in resilience, highlighting the potential of culturally adapted, school-based SEL programs to enhance psychosocial well-being and prevent mental health disorders in vulnerable populations [32]. Similarly, the “Youth First” program, a psychosocial resilience, adolescent health, and gender rights intervention, which aims to promote the psychosocial, physical, and educational wellbeing of its participants, in Bihar, India, improved participants' awareness of personal strengths and problem-solving strategies, suggesting that school-based resilience interventions can empower youth in low- and middle-income countries by enhancing their psychosocial skills [33]. In the context of early childhood, a yoga and mindfulness program for preschoolers in a predominantly Black/African American community in the southeastern US showed significant improvements in social-emotional skills and resilience, as evidenced by higher scores in initiative and self-control [34]. Furthermore, a resilience-focused intervention for at-risk adolescents in a low-income, Latinx immigrant community demonstrated feasibility and acceptability, with participants showing decreased subclinical symptoms and increased positive social attribution bias [35]. These findings collectively underscore the effectiveness of SEL interventions in building resilience among youth, emphasizing the importance of culturally and contextually tailored programs to address the unique challenges faced by different communities. This demonstrates the viability of offering social-emotional programs to youth and adolescents.

One such program is the HEROES program, a social-emotional program designed to improve student resilience. The HEROES program is a strength-based program designed to lead young people through an educational and experiential journey where they discover their dominant strengths, build self-confidence, understand healthy relationships and connect with others, and contribute to the greater good. Specifically, youth who go through the HEROES program are supported to form positive attachments, feel empowered and optimistic about their future, understand that they are an important part of something greater than themselves, know that they are a value and will be treated fairly and equally, clearly understand innate value, know they will be supported to succeed, and learn to change and care for themselves.

## Current study

Despite the theoretical basis of the HEROES program, there has yet to be an empirical evaluation examining the effects of this program on improving student resilience. As such, the goal of this study was to assess the effectiveness of the HEROES program of improving student resilience, self-esteem, and psychological flourishing. In addition, gender was included as a grouping variable to explore whether there are differences. The purpose of the current study is to answer the following research questions:

1. Are there changes in student resilience across time, and does this effect differ by gender?
2. Are there changes in student self-esteem across time, and does this effect differ by gender?
3. Are there changes in student’ flourishing across time, and does this effect differ by gender?

It is expected that participation in the HEROES program will result in increased mean resilience scores across the four time points. Additionally, it is expected that there will be an increase in mean student self-esteem scores and mean flourishing scores across the four time points.

## Method

### Participants and procedure

A purposeful convenience sampling strategy was used to collect data from a rural school in Western Canada. Inclusion criteria for participation required the participants to be in Grade 7 or 8 with parental consent and self-assent to participate in the study, and be able to read, write, and understand English fluently. Students were recruited for the study because their class had enrolled to receive HEROES training. As a result, a letter was sent home to inform parents that their children had the opportunity to participate in the program.

The authors confirm that the research presented in this article was carried out following the ethical guidelines. Upon approval from the research ethics board, informed consent was obtained from all parents/legal guardians with an assent from the participant. Participants were given a pre-survey a week before starting the HEROES program (November 22, 2021). Participants were given a post-survey upon completion of HEROES (April 13, 2022) and two additional post-surveys at approximately two months (June 20, 2022) and five months (September 19, 2022) after the completion of HEROES. Completion of the survey was optional. A longitudinal research design was used which involves tracking a group of individuals over time to evaluate the effects of a program. Longitudinal cohort designs collect data from a cohort of participants at multiple time points, which allows the examination of changes in outcomes over time.

### The HEROES program

The HEROES program was created out of the belief that to build resilience, youth need to be surrounded by individuals that will support them through adolescence, while they are engaging in identity formation. The HEROES program presents students with the idea that everyone has internal strengths (gifts and abilities) that are unique to them. As youth participate in the HEROES program, they learn to recognize their value and develop skills to believe in themselves, choose the right path, and live with purpose. Specifically, youth who go through the HEROES program are supported to form positive attachments, feel empowered and optimistic about their future, understand that they are an important part of something greater than themselves, know that they are a value and will be treated fairly and equally, clearly understand innate value, know they will be supported to succeed, and learn to change and care for themselves.

The HEROES program is designed for youth between the ages of 11 and 15. The curriculum is provided throughout the year across twelve sessions, taking approximately 12–20 h to complete the curriculum. The curriculum can be taken in person or online. The design of the curriculum is centered on leading youth through an educational and experiential journey where they discover their dominant strengths, build self-confidence, understand healthy relationships and connect with others, and contribute to the greater good. The emphasis of the program is strength based. The objective is for each participating youth to go through each module of the program.

Each module (i.e., session) focuses on dimensions of self-discovery (e.g., biological (feeling), psychological (thought), behavioral (experience), and convictional (empowered)). The biological modules emphasize the need to feel safe and value. The psychological modules highlight the need for inspiration and the potential for success. The behavioral modules center on self-awareness of innate strength. Lastly, the convictional modules focus on the understanding how success is generated. Activities in the modules include self-reflection, journaling, and case studies. For example, one activity in the behavioral modules focus on the labels participants assign to themselves, helping students understand and challenge these perceptions. The modules are facilitated by instructors who build confidence, foster positive relationships, and equip participants with essential skills and competencies. Through exploring their strengths and experiential learning opportunities, the goal is to empower students to develop the capacity for positive and sustainable change, leading to resilience and flourishing.

## Measures

### Connor-Davidson resilience scale—10 items (CD-RISC-10)

The CD-RISC-10 is a 10-item scale that measures the basic components of resilience including your abilities, standards, and characteristics; trusting your intuition, enduring hard feelings, and recovering from stress; accepting change positively and having safe relationships; the amount of control you feel you have over your circumstances; and how spirituality influences you [36]. The construct of resilience is measured on a 5-point Likert scale (1 = not at all true, 2 = rarely true, 3 = sometimes true, 4 = often true, 5 = true nearly all the time). Scores range from 0–40, with higher scores indicating greater resilience. It has demonstrated adequate internal consistency of Cronbach’s α = 0.85 [37, 38]. The test–retest correlation was moderate between r = 0.61 and 0.71 depending on the sample [37, 38]. The CDRISC also showed adequate convergent validity with depressive symptoms (r = − 0.51), self-efficacy (r = 0.31), and self-mastery (r = 0.21) [38]. There is evidence that the CD-RISC-10 is acceptable for use with youth (Cronbach’s α = 0.85 [39]

### Rosenberg self-esteem scale (RSES)

The RSES is a 10-item scale that measures components of self-esteem such as self-confidence and self-depreciation [40]. The construct of self-esteem is measured on a 4-point Likert scale (1 = strongly disagree, 2 = disagree, 3 = agree, 4 = strongly agree). Total scores range from 0 to 30, with higher scores indicating higher self-esteem. The RSES has demonstrated adequate internal consistency of Cronbach’s α = 0.77 [40]. The test–retest correlation for a 2 week interval was r = 0.85 [41]. RSES scores were correlated with depression (r = 0.65), anxiety (r = 0.71) and positive view of self (r = − 37) [42] showing adequate convergent validity. This scale has been validated in an adolescent sample (Cronbach’s α = 81) [43].

### Flourishing scale (FS)

The FS is an 8-item scale that measures self-perceived success in areas including purpose, optimism, self-esteem, and relationships [44]. The scale provides a single well-being score. Items are measured on a 7-point Likert scale (1 = strongly disagree, 2 = disagree, 3 = slightly disagree, 4 = neither agree nor disagree, 5 = slightly disagree, 6 = agree, 7 = strongly agree). Scores range from 8 to 56, with higher scores reflecting higher levels of psychological flourishing. The FS has been shown to have good internal consistency of Cronbach’s α = 0.87 [44]. It also has been shown to have good convergent validity with other well-being scales, including the Satisfaction with Life Scale (r = 0.62) and the Life Orientation Test (LOT) (− 0.59) [44]. There is evidence that this scale can be appropriately used on an adolescent population (Cronbach’s α = 0.87) [45].

### Data analyses

Prior to conducting analyses, a power calculation using G*Power 3.1.9.7 [46] was conducted to ensure that there were enough participants to carry out the proposed analyses. Using a medium effect size, α = 0.05, 0.80 power, and correlation among the repeated measures = 0.3, it was determined that 40 participants per gender group were required to conduct the analysis.

Means and standard deviations were calculated from the data for each scale. The mean was used to estimate the average scores for resilience, self-esteem, and flourishing. The standard deviation was used to estimate the variability of resilience, self-efficacy, and flourishing. Both the means and standard deviations were also used to compare different time points to assess whether mean resilience, self-efficacy, and flourishing scores showed significant change after completion of the HEROES program. Repeated-measured analysis of variance (ANOVA) was used to analyze the mean change across multiple time points to identify whether there were changes in mean score across the four time points. To test the assumption of normality, the skew of each of the variables (i.e., resilience, self-esteem, and flourishing) were calculated. Skew values were calculated for each variable and inspected to ensure that there were not large deviations from 0. The skew values for each variable was found to be less than ± 1, indicating that the normality assumption is acceptable allowing the repeated measures ANOVA to be used [47]. Mauchly’s test was used to test the assumption of sphericity for each of the repeated-measures ANOVAs.

## Results

A total of 87 participants (42 males, 41 females, and 3 non-binary youth) completed all 4 surveys between November 2021 and September 2022. Due to the small sample size of non-binary youth and the potential of increased Type I errors resulting from drastically unbalanced group sizes, they were not included in the statistical analyses. Most students were 12 years of age with a range between 11 years old to 13 years old (M = 11.99, SD = 0.36). The ethnic background of students attending this school tend to be primarily of European descent, however, this information was not gathered directly from students.

### Descriptive statistics

Descriptive statistics are shown in Table 1. Means and standard deviations are provided for each of the resilience, self-esteem, and flourishing scales across the four data collection periods (i.e., pre, post, 2 month follow up, and 5 month follow up).

**Table 1:** Descriptive statistics

Variable	Gender	Pre. M (SD)	Post. M (SD)	2 mo. M (SD)	5 mo. M (SD)
Resilience (CD-RISC-10)	Female	23.93 (8.20)	34.26 (8.30)	34.90 (7.48)	39.04 (7.58)
	Male	27.26 (5.88)	37.73 (5.57)	38.35 (6.25)	37.14 (6.94)
	Non-binary	15.33 (7.37)	29.33 (7.37)	28.33 (7.02)	36.00 (6.08)
Self-esteem (RSES)	Female	25.80 (6.75)	26.69 (6.61)	27.66 (5.99)	29.68 (6.07)
	Male	28.42 (6.35)	29.40 (5.87)	29.56 (6.28)	28.10 (5.16)
	Non-binary	20.67 (2.88)	25.33 (4.51)	22.67 (1.53)	25.67 (4.04)
Flourishing (FS)	Female	42.90 (9.56)	45.37 (9.17)	46.31 (12.99)	48.04 (7.79)
	Male	45.17 (7.35)	45.31 (7.11)	46.03 (8.08)	44.93 (7.99)
	Non-binary	37.33 (7.57)	38.67 (6.66)	41.00 (11.14)	41.33 (12.74)

## Main analysis

### Resilience

**Fig. 1:**
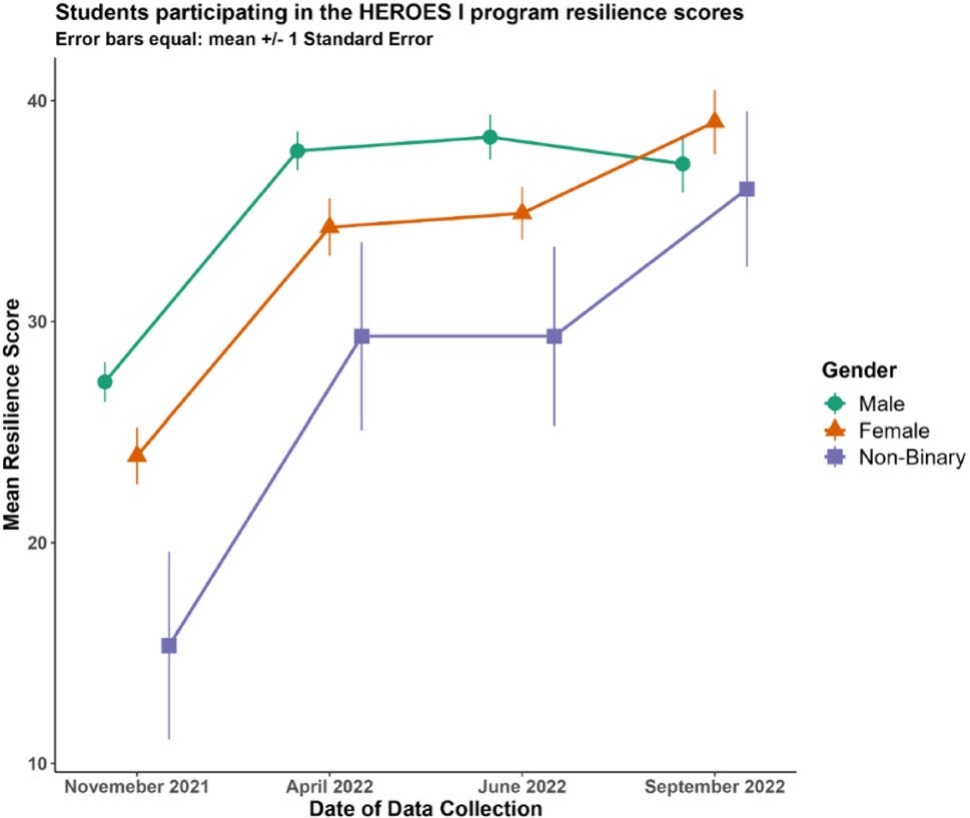
Change in resilience score across the four time points

A repeated-measures ANOVA was conducted to examine the effect of time (pre-HEROES, post-HEROES, 2 months post-HEROES 1, 5 months post-HEROES) and gender (male, female) on resilience. Mauchly’s test indicated that the assumption of sphericity was violated, so degrees of freedom were corrected using Greenhouse–Geisser estimates of sphericity. There was a main effect of time, revealing that there was a significant difference across the different time points, *F*(1, 91) = 42.48, *p* < 0.001, *η*^2^ = 0.46 showing an increase in resilience (see Fig. 1). Post-hoc analyses were conducted using Tukey’s HSD to determine where the significant differences across the time points lie. There was a significant difference between time 1 (Pre-HEROES; M = 25.34) and time 2 (post-HEROES; M = 35.61), *p* < 0.001, time 1 and time 3 (2-months post-HEROES; 36.42), *p* < 0.001, and time 1 and time 4 (5 months post-HEROES; M = 37.74), *p* < 0.001. This indicates that both male and female students showed an increase in resilience immediately after participating in the HEROES program (time point 2). Also, students maintained this increase in resilience at 2 months (time 3) and 5 months (time 4) after participating in the HEROES program.

### Self-Esteem

The second objective of this study was to investigate the change and maintenance of student self-esteem. Using a repeated-measures ANOVA, no significant difference was found across the different time points on the RSES, *F*(3, 153) = 1.91, *p* > 0.05.

### Flourishing

A repeated-measures ANOVA approach was conducted to examine the effect of time and gender on flourishing. There was no significant effect of time across the different time points, *F*(3, 153) = 2.24, *p* > 0.05.

## Discussion

This study aimed to evaluate the effects of the HEROES program on student resilience, self-esteem, and flourishing. The results showed a significant increase in resilience among students after participation. Students also maintained this increase in resilience for two and five months after participating in the HEROES program. There were no significant changes in self-esteem and flourishing after completing the program, or at any follow-up points.

Resilience increased in participants across the four time points of the study. According to Yeager and Dweck [48], resilience is a skill that can be developed and improved with practice, just like any other skill. The HEROES program provided students with practical strategies and techniques for coping with stressors, facing challenges, and overcoming adversity, thus increasing their resilience. This aligns with research that found that changes in adolescent’s social emotional skills were positively and significantly related to changes in resilience [49]. Domitrovich and colleagues found that SEL based programming is a critical factor in the behavioural change process and is central in promoting resilience [50]. Altogether, the findings, along with the current literature, illustrate the important association between teaching youth social emotion skills and increased resilience.

Although statistical analyses in this study were limited to male and female students, the resilience trajectory of non-binary students, as shown in Fig. 1, provides valuable insights into their unique response to the HEROES program. Figure 1 illustrates the changes in resilience average scores across the four time points for male, female, and non-binary students participating in the HEROES program. Non-binary students began with the lowest resilience scores at time 1 and showed substantial improvement over the course of the study. Although their scores remained lower than those of male and female students at each time point, the difference between their scores and those of the other groups decreased substantially by time 4. Notably, by the end of the program, resilience scores across all groups were similar, suggesting that the program effectively fostered resilience across diverse groups. The plot also highlights greater variability in resilience scores for non-binary students, as shown by larger error bars, suggesting heterogeneity within this group and underscoring the importance of tailored approaches to support their unique needs. However, there were only three participants in this group, so the variability estimates may not be accurate. While these observations were not formally analyzed, they highlight the potential of the HEROES program to promote equitable resilience gains and suggest avenues for future research to explore the experiences and outcomes of non-binary students in more depth.

Resilience and self-esteem are related to each other in several ways. When a person is resilient, they can bounce back from setbacks and cope effectively with adversity. This can lead to increased self-esteem because of the feeling of empowerment and capability to handle situations [51, 52]. As such, it was unexpected that self-esteem did not increase in students after participating in the program. There may be several reasons to why the HEROES program did not produce significant findings in self-esteem in this group of participants. In this study, the sample of participants who participated in the HEROES program already had high levels of self-esteem at the beginning of the program. Thus, there may not have been much more room for improvement in self-esteem scores, leading to non-significant findings. Additionally, the impact of other resilience programs on self-esteem has shown mixed results, with a small meta-analysis finding non-significant improvements in self-esteem [53]. As such, more research is necessary to better understand the effect of resilience training on self-esteem and confidence.

The third aim of this study was to understand how students flourishing changed resulting from participating in the HEROES program. No significant changes in flourishing were observed immediately post-intervention or at follow up. These results were unexpected, given the positive relationship between resilience and well-being [54]. One possible explanation for this outcome is the timing of data collection. Previous research suggests that higher resilience enables better stress management, which can lead to improved well-being [55]. As such, it is possible that students had not yet had the chance to practice these skills within the follow up period, explaining the lack of improved flourishing. Furthermore, a systematic review by Abulfaraj, Upsher [56] reported mixed results regarding the impact of resilience programs on well-being, highlighting the need for further research. Future studies should investigate the long-term effects of resilience programs on flourishing to better understand the potential benefits.

The HEROES program equipped students with essential skills and tools to build resilience, producing lasting effects that extended months beyond program completion. The sustained improvements observed in this study suggest that students continue to actively practice the strategies they have learned, leading to further growth, as evidenced five months post-program. Moreover, HEROES aimed to empower students with adaptable skills applicable across various situations. It may be the case that participants are incorporating these skills into other areas of their lives, contributing to ongoing enhancement of their overall well-being. As a result, student reports of increased resilience may be due to consistent practice and application of the program's skills across various contexts. However, more rigorous study designs must be utilized before making this assertion.

## Implications

The findings from this study have notable implications for educational practice, SEL program development, and mental health intervention, alongside clear directions for future research. The observed increase in resilience among students following participation in the HEROES program underscores the value of school-based SEL programs as a proactive mental health intervention. With the unprecedented rise in mental health challenges among youth [57], integrating resilience-focused SEL programs within schools provides a structured approach to equipping students with the skills to navigate stress and adversity. Programs like HEROES thus offers a scalable option for supporting youth well-being and fostering a positive school climate.

Future research should aim to replicate and extend these findings by assessing the program’s impact on larger and more diverse samples. A larger sample size would increase the reliability and accuracy of results, making the findings more representative and generalizable to broader populations. Additionally, evaluating the HEROES program across different demographic groups and school settings will clarify its efficacy and adaptability. Programs like HEROES may vary in effectiveness across diverse populations, so further research is needed to understand how different contexts and backgrounds influence the program’s impact.

The study’s longitudinal component provided valuable insights into the program’s effects over time, with resilience gains maintained at two- and five-month follow-ups. However, conducting a more extended longitudinal analysis after the HEROES program could offer deeper insights into the program’s long-term effects. Tracking changes in key outcomes across several years would allow researchers to analyze how resilience, self-esteem, and flourishing evolve over time, providing essential information on whether and when periodic booster sessions might be beneficial for sustaining program gains. Booster sessions or additional follow-up activities could help reinforce resilience skills, especially as students encounter new stressors or developmental transitions.

Educator involvement is another critical factor that could enhance the effectiveness of SEL programs. Teachers play a crucial role in reinforcing social-emotional learning (SEL) principles by fostering resilience in their classrooms. They create supportive environments through well-established routines, positive language, and encouraging group dialogue [58]. Providing teachers with resilience-building training and integrating SEL into daily interactions may further amplify the HEROES program’s impact. Future research should also explore the role of facilitators more directly, examining how variations in facilitation style, experience, and training affect program outcomes, as it has previously been found that facilitators significantly impact participant outcomes in these types of programs [54]. Understanding these dynamics could inform tailored facilitator training and improve the consistency and effectiveness of SEL program delivery.

In summary, the HEROES program demonstrates promise as a resilience-building intervention in school settings, emphasizing the potential role of SEL programs in promoting mental health and well-being among youth. By pursuing larger, more diverse samples, conducting extended longitudinal analyses, investigating facilitator influences, and incorporating booster sessions, future research can further optimize SEL programs to enhance resilience, self-esteem, and flourishing across varied populations and contexts. Given the association of resilience with better mental health outcomes [24], implementing this program would also likely improve student mental health and well-being.

## Limitations

The study involved a relatively small sample size, specifically targeting Grade 7 and 8 students in a single rural school in Alberta, Canada. The limited sample size and narrow demographic context restrict the generalizability of the findings to other populations, particularly urban, ethnically diverse, or socio-economically varied groups. Furthermore, the exclusion of non-binary participants from statistical analyses due to small sample size further narrows the demographic representation. Additionally,the lack of a control or comparison group is a threat to the internal validity of these results, limiting the ability to infer that changes in participants are due to only program effects.

The study relies on self-report scales for measuring resilience (CD-RISC-10), self-esteem (RSES), and flourishing (FS), which introduces potential biases such as social desirability or self-perception inaccuracies. Self-report measures may not capture the full complexity of participants' experiences and resilience-related behaviors, leading to a limited understanding of the program's effects. The HEROES program showed a significant impact on resilience, but not on self-esteem or flourishing. This could reflect limitations in the study’s measurement tools or insufficient sensitivity in capturing subtle changes in these dimensions. Additionally, baseline self-esteem levels were already high, potentially leading to a ceiling effect where significant improvements are less likely or detectable.

The study does not account for facilitator effects, which may influence program outcomes. Previous research indicates that facilitators can significantly impact SEL program effectiveness depending on their training, experience, and interaction style [54]. Without examining the role of the facilitator, it’s challenging to know whether the observed effects are due to the program content itself or variations in facilitation [59]. As such, it is possible that it is the facilitators that had significant effects on participants, rather than the program.

## Conclusion

This study resulted in a clearer understanding of the outcomes and effectiveness of the HEROES program in equipping students with tools to enhance their resilience. Findings from this study provide insight into student outcomes and experiences of the HEROES program. Participants experienced an increase in resilience immediately after completing the HEROES program, and they also maintained high levels of resilience at 2- and 5 months after program completion. Also, participants showed some change in confidence and self-esteem post-HEROES, although this change was not significant. Altogether, the HEROES program may be a beneficial program for schools to implement to improve student resilience. Further research is needed to better understand the impact of the HEROES program on self-esteem and flourishing.

## Data Availability

The datasets generated during and/or analyzed during the current study are available from the corresponding author on reasonable request.

## References

[1] Abramson A, Children’s mental health is in crisis. 2022: APA.org . https://www.apa.org/monitor/2022/01/special-childrens-mental-health.

[2] Varadkar SM, Gadgil P. Closing the mental health gap to support good life chances for children and young people. Lancet Glob Health. 2024. 10.1016/s2214-109x(23)00519-3. 37980912 10.1016/S2214-109X(23)00519-3

[3] Eapen V, et al. Stemming the tide of mental health problems in young people: challenges and potential solutions. Aust N Z J Psychiatry. 2023;57(4):482–8. 10.1177/00048674221136037. 36377648 10.1177/00048674221136037

[4] Brooks R. Children at risk: fostering resilience and hope. Am J Orthopsychiatry. 1994;64(4):545–53. 7847570 10.1037/h0079565

[5] Morales-Muñoz I, et al. Impact of anxiety and depression across childhood and adolescence on adverse outcomes in young adulthood: a UK birth cohort study. Br J Psychiatry. 2023;222(5):212–20. 10.1192/bjp.2023.23. 36919351 10.1192/bjp.2023.23 PMC10895507

[6] Göbel K, et al. Co-occurrence, stability and manifestation of child and adolescent mental health problems: a latent transition analysis. BMC Psychol. 2022. 10.1186/s40359-022-00969-4. 36376939 10.1186/s40359-022-00969-4 PMC9664619

[7] Resnick MD. Protective factors, resiliency and healthy youth development. Adolesc Med. 2000;11(1):157–65. 10640344

[8] McGillivray C., Pidgeon A. Resilience attributes among university students: a comparative study of psychological distress, sleep disturbances and mindfulness. Eur Sci J ESJ. 2015. 11(5); https://eujournal.org/index.php/esj/article/view/5174.

[9] Talaie M, Mohammadi Y, Raeisoon M. The effects of resilience training on mental health among students. Mod Care J. 2024. 10.5812/mcj-143805.

[10] Elias MJ, Weissberg RP. Primary prevention: educational approaches to enhance social and emotional learning. J Sch Health. 2000;70(5):186–90. 10.1111/j.1746-1561.2000.tb06470.x. 10900595 10.1111/j.1746-1561.2000.tb06470.x

[11] Torre D, Murphy J. Communities of parental engagement: new foundations for school leaders’ work. Int J Leadersh Educ. 2016;19(2):203–23.

[12] Baird S, et al. Accelerating well-being for adolescents through transformative public policy: a framework for action. J Adolesc Health. 2024;75(4Supplement):S37–46. 39293876 10.1016/j.jadohealth.2024.03.013 PMC11825381

[13] Karumazondo JJ, et al. Innovative professional development for multisectoral policy making and programming for adolescent wellbeing. J Adolesc Health. 2024;74(4):637–43. 10.1016/j.jadohealth.2023.12.025. 38323967 10.1016/j.jadohealth.2023.12.025

[14] American Psychological Association. Resilience. In: APA dictionary of psychology. https://dictionary.apa.org/resilience. Accessed 4 Feb 2025.

[15] Masten A. Ordinary magic: Resilience processes in development. Am Psychol. 2001;56(3):227–38. 11315249 10.1037//0003-066x.56.3.227

[16] Maunder RG, et al. Relationship between three aspects of resilience—adaptive characteristics, withstanding stress, and bouncing back—in hospital workers exposed to prolonged occupational stress during the COVID-19 pandemic: a longitudinal study. BMC Health Ser Res. 2023. 10.1186/s12913-023-09731-x. 10.1186/s12913-023-09731-x PMC10303818 37380994

[17] Cheng C, Chen S. Unmasking resilience in the ‘new normal’: coping with unprecedented stressors amid COVID-19. Curr Opin Behav Sci. 2024;55: 101346.

[18] Panagou C, Macbeth A. Trajectories of risk and resilience: the role of empathy and perceived social support in the context of early adversity. Child Abuse Negl. 2024;153: 106811. 38703490 10.1016/j.chiabu.2024.106811

[19] Buzzanell PM, Houston JB. Communication and resilience: multilevel applications and insights—a journal of applied communication research forum. J Appl Commun Res. 2018. 10.1080/00909882.2017.1412086.

[20] Fenwick-Smith A, Dahlberg EE, Thompson SC. Systematic review of resilience-enhancing, universal, primary school-based mental health promotion programs. BMC Psychol. 2018. 10.1186/s40359-018-0242-3. 29976252 10.1186/s40359-018-0242-3 PMC6034212

[21] Zhan Y, Hong JC, Zhao L. Effects of goal achievement motivation on K-12 students’ resilience in STEAM activities: the mediating role of in-school social supports. J Res Technol Educ. 2024. 10.1080/15391523.2024.2378073.

[22] Wyman PA, et al. The role of children’s future expectations in self-system functioning and adjustment to life stress: a prospective study of urban at-risk children. Dev Psychopathol. 1993;5(4):649–61.

[23] Ashton K, et al. Adult support during childhood: a retrospective study of trusted adult relationships, sources of personal adult support and their association with childhood resilience resources. BMC Psychol. 2021. 10.1186/s40359-021-00601-x. 34176519 10.1186/s40359-021-00601-x PMC8237477

[24] Barankin T, Khanlou N. Growing up resilient: ways to build resilience in children and youth. Cen Addictn Ment Health. 2007;18(4):357.

[25] Sapienza J, Masten A. Understanding and promoting resilience in children and youth. Curr Opin Psychiatry. 2011;24(4):267–73. 21546838 10.1097/YCO.0b013e32834776a8

[26] Kichler JC, Kaugars AS. Topical review: applying positive development principles to group interventions for the promotion of family resilience in pediatric psychology. J Pediatr Psychol. 2015;40(9):978–80. 10.1093/jpepsy/jsu115. 25577789 10.1093/jpepsy/jsu115

[27] Kilpatrick KD, et al. An evaluation of the potential efficacy and feasibility of the resilience education program: a tier 2 internalizing intervention. Sch Ment Heal. 2021;13(2):376–91. 10.1007/s12310-021-09428-8.

[28] Durlak JA, et al. The impact of enhancing students’ social and emotional learning: a meta-analysis of school-based universal interventions. Child Dev. 2011;82(1):405–32. 10.1111/j.1467-8624.2010.01564.x. 21291449 10.1111/j.1467-8624.2010.01564.x

[29] Masten AS, Tellegen A. Resilience in developmental psychopathology: contributions of the project competence longitudinal study. Dev Psychopathol. 2012;24(2):345–61. 22559118 10.1017/S095457941200003X

[30] Al-Hroub A, Al-Hroub R. Empowering the vulnerable: the impact of sel on traumatized children’s academic and social outcomes in crises. Curr Psychiatry Rep. 2024;26(12):777–81. 10.1007/s11920-024-01555-8. 39523250 10.1007/s11920-024-01555-8 PMC11706873

[31] Rudolph KD, et al. Cultivating emotional resilience in adolescent girls: Effects of a growth emotion mindset lesson. Child Dev. 2025;96(1):389–406. 10.1111/cdev.14175. 39367719 10.1111/cdev.14175 PMC11693836

[32] Seff I, et al. Supporting social emotional learning and wellbeing of displaced adolescents from the middle east: a pilot evaluation of the ‘forward with peers’ intervention. BMC Psychiatry. 2024. 10.1186/s12888-024-05544-2. 38438860 10.1186/s12888-024-05544-2 PMC10910802

[33] Leventhal KS, et al. Promoting wellbeing and empowerment via youth first: exploring psychosocial outcomes of a school-based resilience intervention in Bihar. India Frontiers in Psychiatry. 2022. 10.3389/fpsyt.2022.1021892. 36465290 10.3389/fpsyt.2022.1021892 PMC9712804

[34] Bazzano AN, et al. Yoga and mindfulness for social-emotional development and resilience in 3–5 year-old children: non-randomized, controlled intervention. Psychol Res Behav Manag. 2023. 10.2147/prbm.s385413. 36660255 10.2147/PRBM.S385413 PMC9844140

[35] Clauss JA, et al. Development of a transdiagnostic, resilience-focused intervention for at-risk adolescents. J Ment Health. 2023;32(3):592–601. 36369940 10.1080/09638237.2022.2140790 PMC10175511

[36] Connor KM, Davidson JRT. Development of a new resilience scale: the Connor-Davidson resilience scale (CD-RISC). Depress Anxiety. 2003;18(2):76–82. 10.1002/da.10113. 12964174 10.1002/da.10113

[37] Notario-Pacheco B, et al. Reliability and validity of the Spanish version of the 10-item Connor-Davidson Resilience Scale (10-item CD-RISC) in young adults. Health Qual Life Outcomes. 2011;9(1):63. 10.1186/1477-7525-9-63. 21819555 10.1186/1477-7525-9-63 PMC3173284

[38] Tourunen A, et al. Psychometric properties of the 10-item Connor-Davidson resilience scale among Finnish older adults. Aging Ment Health. 2021. 10.1080/13607863.2019.1683812. 31703533 10.1080/13607863.2019.1683812

[39] Nartova-Bochaver S, Korneev A, Bochaver K. Validation of the 10-item Connor-Davidson resilience scale: the case of Russian youth. Front Psych. 2021. 10.3389/fpsyt.2021.611026. 10.3389/fpsyt.2021.611026 PMC7902788 33643092

[40] Rosenberg M. Rosenberg self-esteem scale (RSES) [database record]. APA PsycTests: Washington, D.C; 1965.

[41] Silber E, Tippett JS. Self-esteem: clinical assessment and measurement validation. Psychol Rep. 1965. 10.2466/pr0.1965.16.3c.1017.5826497

[42] Cooper-Evans S, et al. Self-esteem as a predictor of psychological distress after severe acquired brain injury: an exploratory study. Neuropsychol Rehabil. 2008;18(5–6):607–26. 10.1080/09602010801948516. 18609016 10.1080/09602010801948516

[43] Moksnes UK, et al. Validation of Rosenberg self-esteem scale among Norwegian adolescents—psychometric properties across samples. BMC Psychol. 2024. 10.1186/s40359-024-02004-0. 39334492 10.1186/s40359-024-02004-0 PMC11437639

[44] Diener E, et al. New well-being measures: short scales to assess flourishing and positive and negative feelings. Soc Indic Res. 2010;97(2):143–56. 10.1007/s11205-009-9493-y.

[45] Romano I, et al. Measurement invariance of the flourishing scale among a large sample of Canadian adolescents. Int J Environ Res Public Health. 2020;17(21):7800. 33113772 10.3390/ijerph17217800 PMC7663739

[46] Faul F, et al. G*power 3: a flexible statistical power analysis program for the social, behavioral, and biomedical sciences. Behav Res Methods. 2007;39(2):175–91. 17695343 10.3758/bf03193146

[47] Tabachnick B, Fidell L. Using multivariate statistics. 7th ed. London: Pearson Education; 2019.

[48] Yeager DS, Dweck CS. Mindsets that promote resilience: when students believe that personal characteristics can be developed. Educ Psychol. 2012. 10.1080/00461520.2012.722805.

[49] Martinsone B, et al. Adolescent social emotional skills, resilience and behavioral problems during the COVID-19 pandemic: a longitudinal study in three European countries. Front Psych. 2022. 10.3389/fpsyt.2022.942692. 10.3389/fpsyt.2022.942692 PMC9376252 35978848

[50] Domitrovich CE, et al. Social-emotional competence: an essential factor for promoting positive adjustment and reducing risk in school children. Child Dev. 2017;88(2):408–16. 10.1111/cdev.12739. 28213889 10.1111/cdev.12739

[51] McGee P. Self-confidence: the remarkable truth of how a small change can boost your resilience and increase your success. Hoboken: John Wiley & Sons; 2020.

[52] Veselska Z, et al. Self-esteem and resilience: the connection with risky behavior among adolescents. Addict Behav. 2009;34(3):287–91. 10.1016/j.addbeh.2008.11.005. 19056183 10.1016/j.addbeh.2008.11.005

[53] Leppin AL, et al. The efficacy of resiliency training programs: a systematic review and meta-analysis of randomized trials. PLoS ONE. 2014;9(10): e111420. 10.1371/journal.pone.0111420. 25347713 10.1371/journal.pone.0111420 PMC4210242

[54] Arifia R, Amalia S. A Literature Review: Relationship between Resilience And Well-Being Among Adolescents and Early Adults. Scientia. 2024. 10.51773/sssh.v3i1.258.

[55] Calonia J, et al. Exploring well-being in college students: the influence of resilience and Social Support. Int J Innov Sci Res Technol (IJISRT). 2024;9(5):3481–91.

[56] Abulfaraj GG, et al. The impact of resilience interventions on university students’ mental health and well-being: a systematic review. Educ Sci. 2024;14(5):510. 10.3390/educsci14050510.

[57] Wiens K, et al. A growing need for youth mental health services in Canada: examining trends in youth mental health from 2011 to 2018. Epidemiol Psychiatric Sci. 2020. 10.1017/s2045796020000281. 10.1017/S2045796020000281 PMC7214527 32299531

[58] Nolan A, Taket A, Stagnitti K. Supporting resilience in early years classrooms: the role of the teacher. Teach Teach. 2014;20(5):595–608. 10.1080/13540602.2014.937955.

[59] Deli W, Kaur A, Awang Hashim R. WHO delivers it and how it is delivered: effects of social-emotional learning interventions on learning anxiety and dropout intention. Malays J Learn Instr. 2021. 10.32890/mjli2021.18.1.1.

